# Adverse environmental conditions influence age-related innate immune responsiveness

**DOI:** 10.1186/1742-4933-6-7

**Published:** 2009-05-30

**Authors:** Linda May, Anita HJ van den Biggelaar, David van Bodegom, Hans J Meij, Anton JM de Craen, Joseph Amankwa, Marijke Frölich, Maris Kuningas, Rudi GJ Westendorp

**Affiliations:** 1 Department of Gerontology and Geriatrics, Leiden University Medical Center, Leiden, the Netherlands; 2 Cell Biology Division, Telethon Institute for Child Health Research, Centre for Child Health Research, The University of Western Australia, Perth, Australia; 3 Ghana Health Service, Upper East region, Bolgatanga, Ghana; 4 Department of Clinical Chemistry, Leiden University Medical Center, Leiden, the Netherlands; 5 Netherlands Consortium for Healthy Ageing, Leiden, the Netherlands

## Abstract

**Background-:**

The innate immune system plays an important role in the recognition and induction of protective responses against infectious pathogens, whilst there is increasing evidence for a role in mediating chronic inflammatory diseases at older age. Despite indications that environmental conditions can influence the senescence process of the adaptive immune system, it is not known whether the same holds true for the innate immune system. Therefore we studied whether age-related innate immune responses are similar or differ between populations living under very diverse environmental conditions.

**Methods-:**

We compared cross-sectional age-related changes in *ex vivo *innate cytokine responses in a population living under affluent conditions in the Netherlands (age 20–68 years old, n = 304) and a population living under adverse environmental conditions in Ghana (age 23–95 years old, n = 562).

**Results-:**

We found a significant decrease in LPS-induced Interleukin (IL)-10 and Tumor Necrosis Factor (TNF) production with age in the Dutch population. In Ghana a similar age-related decline in IL-10 responses to LPS, as well as to zymosan, or LPS plus zymosan, was observed. TNF production, however, did not show an age-associated decline, but increased significantly with age in response to co-stimulation with LPS and zymosan.

**Conclusion-:**

We conclude that the decline in innate cytokine responses is an intrinsic ageing phenomenon, while pathogen exposure and/or selective survival drive pro-inflammatory responses under adverse living conditions.

## Introduction

The innate immune system plays a key role in the first line recognition and clearance of pathogens, whilst orchestrating down-stream adaptive immune responses. An adequate innate immune response consists of a delicate balance between pro-inflammatory responses that facilitate pathogen clearance, and counteracting anti-inflammatory responses that control excessive systemic inflammatory host responses [1-4]. Ageing is associated with an impaired capacity of the innate immune system to produce pro- as well as anti-inflammatory cytokines [5,6], which weakens the ability to respond to infections and cancers and has been associated with increased mortality in the elderly [7]. Besides genetic and intrinsic factors, this senescence process of the innate immune system could be environmentally driven.

As far as the adaptive immune system is concerned, there are indications that a high versus low exposure to infectious pathogens influences the ability to produce type-1 and type-2 T helper cell cytokine responses [8]. In addition, there is accumulating evidence that chronic infections, such as with CMV [9], contribute to the age-related decline in T cell cytokine responses. Besides changes in the T cell compartment itself [10], alterations in the innate immune system may be responsible for this observed variation in T cell responsiveness. There are indications that chronic infections, such as with helminths, can modulate Toll-like receptor (TLR)-mediated innate immune responses [11,12], and mouse studies have provided evidence for a decline in innate immune responses with increasing age [13,14]. However, the role of environmental conditions in age-related changes in innate immune responses has not been assessed before. We suggest that in line with observations for T cell responses, persistent immune challenges will accelerate the senescence of the innate immune system in populations experiencing lifelong exposure to infections. However, as we have hypothesized previously, infectious pressure may not allow lower immune responsiveness, and this enhanced age-related decline in innate immune responses may for that reason be distorted by a selective survival of individuals producing strong pro-inflammatory immune responses [15].

The aim of this study was to compare differences in cross-sectional age-related changes in inflammatory immune responses in two populations living under very different environmental conditions: one population born and raised under affluent conditions in the Netherlands; the other population living under lifelong strong adverse conditions in a remote area of the Garu-Tempane district in Ghana. For this reason we studied the production capacity of *ex vivo*-induced levels of the pro-inflammatory cytokine Tumor Necrosis Factor (TNF) and anti-inflammatory cytokine Interleukin (IL)-10 in both populations.

## Results

The Dutch study population consisted of 304 adults, of which 138 were male and 166 were female, with an age-range of 20–68 years (median age is 35 years). The Ghanaian study population consisted of 562 adults, 59 males and 503 females, and with an age-range of 23–95 years (median age is 48 years) were significantly older than the Dutch study population (p < 0.001).

In the Dutch study population the *ex vivo *LPS-induced production of TNF (median is 6878, pg/ml; IQR 5379 – 9431) and IL-10 (median is 2201 pg/ml; IQR 1776 – 2703) was significantly lower compared to the Ghanaian population (TNF: median is 12532 pg/ml; IQR 7789 – 18979, p < 0.001) (IL-10: median is 4370 pg/ml; IQR 3151 – 5967, p < 0.001)). In addition, in response to zymosan or LPS/zymosan co-stimulation the Ghanaian population produced median TNF levels of 12599 pg/ml (IQR 8504 – 18281) and 14597 pg/ml (IQR 8704 – 20757), and median IL-10 levels of 210 pg/ml (IQR 121 – 360) and 581 pg/ml (IQR 354 – 941), respectively.

**Figure 1:**
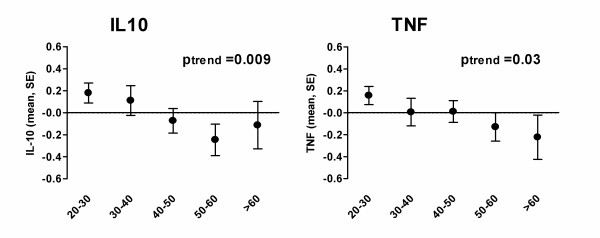
**Age-related pro- (TNF) and anti-inflammatory (IL-10) cytokine responses in the Dutch study population (n = 304)**. Data represent cytokine production upon *ex vivo *stimulation with LPS and are expressed as z-scores with standard errors indicating the deviance from the population mean (zero-value). P-values indicate a trend in cytokine production over age.

**Figure 2:**
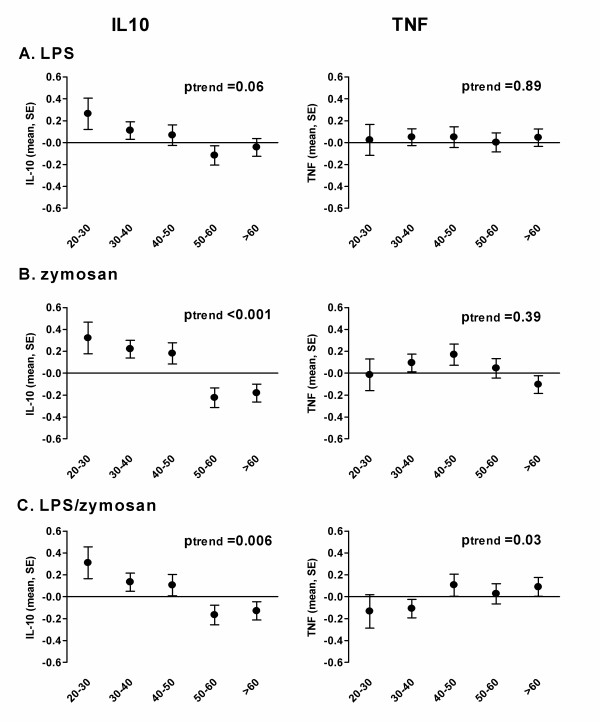
**Age-related pro- (TNF) and anti-inflammatory (IL-10) cytokine responses in the Ghanaian study population (n = 562)**. Data represent cytokine production upon *ex vivo *stimulation with LPS and are expressed as z-scores with standard errors indicating the deviance from the population mean (zero-value). P-values indicate a trend in cytokine production over the age.

A significant age-related decrease in *ex vivo *LPS-induced production of both IL-10 and TNF was observed in the Dutch study population (Table 1 and Figure 1). Similarly, among Ghanaian adults *ex vivo *LPS-induced IL-10 production decreased with age (Table 1 and Figure 2). This was, however, not observed for LPS-induced TNF production, which remained unchanged with increasing age. The age-related decrease in IL-10 responses in the Ghanaian study population was not different to that in the Dutch population (p for interaction = 0.202), whereas that of TNF was (p-int = 0.067). Also in response to zymosan and LPS/zymosan stimulation, IL-10 production significantly decreased with age in the Ghanaian study adults. TNF production remained unchanged in response to zymosan but increased significantly in response to LPS/zymosan co-stimulation (Table 1 and Figure 2).

**Table 1:** Age-dependent decrease of cytokine production

	Dutch		Ghanaian	
	(n = 304)	p-value	(n = 562)	p-value
Change in IL-10 production per 10 years of age (SE)				
LPS	-0.117 (0.045)	0.009	-0.053 (0.029)	0.064
Zymosan	n.d.		-0.116 (0.029)	< 0.001
LPS/zymosan	n.d.		-0.081 (0.029)	0.006
Change in TNF production per 10 years of age (SE)				
LPS	-0.089 (0.041)	0.030	-0.004 (0.028)	0.89
Zymosan	n.d.		-0.025 (0.029)	0.39
LPS/zymosan	n.d.		0.067 (0.030)	0.026

All analyses were adjusted for sex. Estimates indicate the increase in z-scores with standard error per 10 years of age. n.d. not determined

As males were underrepresented in the Ghanaian study population we repeated our analysis by selecting for women only. In line with findings for the total study population, IL-10 production decreased with age in response to all stimuli (LPS: -0.061 SD per 10 years, (SE = 0.029), p = 0.039; zymosan: -0.129 SD per 10 years (SE = 0.029), p < 0.001; LPS/zymosan: -0.101 SD per 10 years (SE = 0.030), p = 0.001) in the group of Ghanaian women, and similar patterns were observed for TNF production (LPS: -0.002 SD per 10 years (SE = 0.029), p = 0.94; zymosan: -0.024 SD per 10 years (SE = 0.030), p = 0.43; LPS/zymosan: 0.064 SD per 10 years (SE = 0.031), p = 0.039). Also for women in the Dutch study population, similar patterns were observed as in the total population: IL-10 changed -0.161 SD per 10 years (SE = 0.061) (P = 0.009) and TNF changed -0.121 SD per 10 years (SE = 0.051) (p = 0.020). Given the differences in age-range in the Dutch and the Ghanaian study population, we repeated the analyses of the Ghanaian sample, restricting to participants for the same age range as in the Dutch sample (22 to 68 years). Here similar results were observed for IL10 production upon stimulation with LPS (-0.100 [0.036], p = 0.005), zymosan (-0.199 [0.035], p < 0.001) and LPS/zymosan (-0.154 [0.036], p < 0.001). Also for TNF similar results were observed upon stimulation with LPS (0.005 [0.036], p = 0.89), zymosan (-0.026 [0.037], p = 0.48) and LPS/zymosan (0.067 [0.038], p = 0.077).

## Discussion

In this study we have shown that in an adult Dutch population that lived under affluent conditions for their entire life, there is a gradual decline in the production of *ex vivo *LPS-induced anti-inflammatory IL-10 as well as pro-inflammatory TNF response with increasing age. A similar decline in IL-10 production was observed in the Ghanaian adults that have experienced a lifelong exposure to infectious pathogens. In contrast TNF responses to LPS remained unchanged and in response to co-stimulation with LPS and zymosan TNF production even significantly increased with age among Ghanaians.

There are several possible explanations for these data, as an age-related decline in the cytokine production capacity of the innate immune system may be intrinsically regulated, environmentally driven or be a result of selective survival. First, we believe that in the Dutch study population that lives in an affluent environment with low infectious exposure and where mortality rates up to the age of 80 years are low, intrinsic age-related effects play a pivotal role and explain the age-related decline in cytokine production. This includes an age-related lower expression of Toll-like receptors [16] and impaired function of all cells of the innate immune system [17-19] that together results in a lower production capacity of cytokines. We propose that the same intrinsic mechanism of senescence of the innate immune system would act in the Ghanaian population.

Second, it is possible that in the Ghanaian population continuous pathogen exposure accelerates ageing of the innate immune system. Although this hypothesis is yet to be proven, there are several indications that chronic infections can modulate innate immune function, including findings that chronic helminthic infections reduce TLR2 expression[11] and our own observations that LPS-induced TNF and IL-10 responses were significantly enhanced in the Ghanaian compared to Dutch study populations. Interestingly, in patients with chronic Hepatitis C Virus (HCV) infections, the over-production of pro-inflammatory cytokines, in particular TNF has been shown to be likely due to a loss of TLR tolerance, a protective mechanism usually in place to limit inflammation[20]. In mice it has been shown that with age this tolerance process is attenuated[13]. Considering that TNF responses remained unchanged or increased with age in the Ghanaian population, we propose that if anything, our data do not support the hypothesis that a lifelong exposure to infections accelerates the age-related decline in innate immune responses, but on the contrary may drive pro-inflammatory responses.

Third, the age-related changes in cytokine production observed in the Ghanaian population may reflect the selective survival of individuals with immune responses that promote survival in adverse environmental conditions [21,2,22]. Previously, we have hypothesized that under adverse conditions people with enhanced pro-inflammatory immune responses may have greater survival potential than those with stronger anti-inflammatory responses [15]. This may explain why IL-10 but not TNF responses decline with age in Ghana.

Inflammation has been suggested to be one of the mechanisms underlying pathogenesis of several age-associated diseases such as cardiovascular disease [23]. Considering the emerging epidemic of chronic diseases in low-income countries [24], we therefore propose that this could well be explained by the here observed increase in pro-inflammatory versus decrease in anti-inflammatory responses in older age groups resulting from overstimulation or selective survival for this pro-inflammatory response pattern.

To our knowledge this is the first study looking at age-related changes in innate immune responses in populations aging under very diverse environmental conditions. A drawback of this study is that for the Dutch study population we did not have data in response to TLR ligands other than LPS. However, given the same patterns to different ligands in Ghana, we would expect no remarkable differences. In further research it should be tested whether the same trends will be observed in the Dutch population. Also age ranges were not completely alike including participants with a larger age-range in the Ghana population than in the Dutch cohort. Also there might be some uncertainty concerning the age of the participants in the highest age-category in the Ghanaian population, as these were perceived ages estimated based on face-value, life-history and mobility. We therefore grouped them as 60 plus. In addition, due to the cross-sectional nature of the study, we can not draw any final conclusions whether age-related changes in cytokine production is indeed an intrinsic phenomenon occurring over age, a result of selective survival and/or pathogen exposure.

In conclusion, in this study we demonstrated for the first time that in both in affluent and adverse environmental conditions there is an age-related decline in the IL-10 production capacity of the innate immune system. For TNF production, a similar decline was observed under affluent environmental conditions, but not under adverse environmental conditions. Lower production of cytokines seems an intrinsic phenomenon of the ageing process whereas chronic infections and/or selective survival may drive cytokine production towards pro-inflammatory responsiveness.

## Methods

### Populations

The Ghanaian part of our study was conducted in the remote Garu-Tempane district in the Upper-East region of Ghana. This densely populated agricultural area is inhabited by several tribes, mostly Bimoba and Kusasi. The Ghana Upper-East region, and especially the Garu-Tempane district, is underdeveloped, poor and mortality rates are high, with main causes of death including malaria, diarrhoea and poor nutrition [25,26]. The vast majority of the people are farmers and the total agricultural process is done by hand labor. In 2001, we mapped the research area using a GPS system[27]. Since 2002, we have revisited the area annually to assess population changes. In 2006 a series of whole-blood assays were taken from a subset of the population. The Dutch study population consisted of subjects enrolled in a study on heritability of cytokine production in twins[28]. All people were born and raised in the Netherlands. A main difference between the general Ghanaian and Dutch population is mortality rates, that in the Netherlands results in a demographic composition with a median age is 39 years[29], whereas in the Garu-Tempane district this is 14 years. The Medical Ethical Committee of the Ghana Health Service, as well as the Medical Ethical Committee of the Leiden University Medical Center approved the studies. Witnessed observed informed consent was obtained from all Ghanaian participants and written informed consent was obtained from the Dutch participants.

### Whole blood stimulation assay and cytokine production

In both of these populations pro-inflammatory and anti-inflammatory cytokine production capacity was assessed by stimulating *ex vivo *whole blood samples with lipopolysaccharide (LPS) as described elsewhere[30,31]. All venous blood samples were drawn in the morning to exclude circadian variation, diluted twofold with RPMI-1640, and within two hours after collection were cultured with medium alone or with an optimal dose of 10 μg/ml *E. coli*-derived LPS (Sigma Aldrich, Zwijndrecht, the Netherlands) in 24-well plates at duplicate volumes of 1 ml for 24 hours in 37°C incubators. In the Ghanaian sample, additional *ex vivo *whole blood stimulations were performed with 100 μg/ml zymosan and with a combination of 10 ng/ml LPS and 100 μg/ml zymosan (Sigma-Aldrich, Schnelldorf, Germany). Procedures and conditions were kept similar in both settings, except that a CO_2 _incubator set at 5% was used in the Netherlands, and ambient CO_2 _levels were induced by a candle jar incubation system in Ghana[32]. In the candle jar incubation system culture plates are placed in an airtight container with a burning candle enclosed, and transferred as a whole to a 37°C incubator once the candle has faded. The compatibility of both systems was compared in a small experiment in which whole blood assays were performed for the same five staff members at both study sites: LPS-induced levels of TNF and IL-10 were comparable for the ambient CO_2 _conditions in Ghana and the incubator set 5% CO_2 _conditions in the Netherlands (data not shown). Supernatants were collected and kept at -20°C in Ghana until transported on dry ice to the Netherlands.

In the Netherlands all samples were stored at -80°C until cytokine levels were determined by ELISA. Cytokine ELISA for human TNF and IL-10 were performed according to manufacturers' guidelines (Central Laboratory of the Blood Transfusion Service, Amsterdam, the Netherlands), with detection limits of 4.0 pg/ml and 3.0 pg/ml respectively.

### Statistical analysis

All cytokine levels were ln-transformed, since they were not normally distributed, and converted into z-scores ((individual level – mean level)/SD), which were used in all analyses. Associations between cytokine responses and age categories of 10 years as a continuous independent variable were assessed with sex adjusted linear regression. Calculations were performed with SPSS version 14.0 (SPSS Inc., Chicago, Illinois, USA).

## Abbreviations

IL-10: Interleukin-10; TNF: Tumour Necrosis Factor; LPS: lipopolysaccharide; SE: standard error.

## Competing interests

The authors declare that they have no competing interests.

## Authors' contributions

LM, AvdB, JM, AdC, JA, MF and RW designed the study. LM, DvB, JM performed fieldwork and whole blood assays in Ghana. AdC collected data from the Dutch Twin study. MF carried out ELISA assays. LM, AvdB and MK analyzed the data. LM, AvdB, MK, DvB and RW wrote the paper. All authors approved the final version of the manuscript.
